# Prediction of freak waves from buoy measurements

**DOI:** 10.1038/s41598-024-66315-3

**Published:** 2024-07-18

**Authors:** Thomas Breunung, Balakumar Balachandran

**Affiliations:** Department of Mechanical Engineering, University of Maryland, College Park, MD 20742 USA

**Keywords:** Rogue waves, Extreme events, Machine learning, Forecasting, Ocean waves, Buoy data, Ocean sciences, Engineering

## Abstract

Freak or rogue waves are a danger to ships, offshore infrastructure, and other maritime equipment. Reliable rogue wave forecasts could mitigate this risk for operations at sea. While the occurrence of oceanic rogue waves at sea is generally acknowledged, reliable rogue wave forecasts are unavailable. In this paper, the authors seek to overcome this shortcoming by demonstrating how rogue waves can be predicted from field measurements. An extensive buoy data set consisting of billions of waves is utilized to parameterize neural networks. This network is trained to distinguish waves prior to an extreme wave from waves which are not followed by an extreme wave. With this approach, three out of four rogue waves are correctly predicted 1 min ahead of time. When the advance warning time is extended to 5 min, it is found that the ratio of accurate predictions is reduced to seven out of ten rogue waves. Another strength of the trained neural networks is their capabilities to extrapolate. This aspect is verified by obtaining forecasts for a buoy location that is not included in the networks’ training set. Furthermore, the performance of the trained neural network carries over to realistic scenarios where rogue waves are extremely rare.

## Introduction

Rogue waves, which are waves larger than waves corresponding to an average sea state, continue to endanger offshore infrastructure and ships (e.g. the recently publicized incident^1^). Indeed, the loss of super-carriers^2^, destruction of offshore equipment^3^, and injuries to sailors and passengers^4,5^ have been linked to occurrences of rogue waves. Although this danger has inspired significant research efforts, the emergence of a rogue wave remains unpredictable.

The unpredictability of rogue waves can be underpinned by assuming that the wave formation is caused by a superposition of elementary waves with unknown phases. According to this popular theory, constructive interference of several elementary waves, which are either modelled with single harmonics or a more complex functions results in the formation of a large wave^6–8^. To observe such a superposition, the phases of the individual waves need to the appropriately synchronized. However, state-of-the-art, operational ocean models^9,10^ do not yield information on the wave phases. Rather, it is generally acknowledged that it is impossible to determine the phases of ocean waves, and rather a random distribution of the phases is assumed^12^. Thus, in this setting, rogue waves are inherently unpredictable. Indeed, phase resolving ocean wave models have been identified as being crucial for predicting the emergence of rogue waves^13^.

On the other hand, rogue waves can be formed by modulation instabilities, most prominently, the Benjamin-Feir instability^2,7,14–16^. Within this mechanism, a single wave train becomes unstable under the addition of appropriately selected modulations. As this instability develops on a slow time scale, in principle, one could, at least in principle, predict the occurrence of this instability with significance advance time. However, most observations of the Benjamin-Feir instability are based on either simplified equations, such as the nonlinear Schrödinger equation and its extensions^17^, or idealized wave tank experiments^18–20^. Due to the lack of conclusive evidence for developing modulation instabilities under realistic conditions, the relevance of this instability for the real ocean has been questioned^6,21,22^.

Leaving aside theoretical discussions on rogue wave formation mechanisms, researchers have proposed practical approaches to forecast rogue waves. For example, average sea parameters such as significant wave height, peak period, and skewness have been related to rogue wave occurrences^23–25^. In general, the excess kurtosis can be most directly linked to extreme waves^26,27^, and this parameter is utilized within the core of an operational rogue wave forecast^28^. However, researchers have pointed out that a high kurtosis is an effect of extreme waves and this does not necessarily indicate causality^21,23,29^. Moreover, the Benjamin-Feir index can be computed from wave spectra^26^, and in principle, one can predict rogue waves caused by this instability type. However, two extensive analyses on available buoy data^21,30^ have demonstrated that all these indicators do not correlate with rogue wave occurrences in practice. More recently, a data-driven strategy to forecast individual waves has been proposed and real-time forecasts of two rogue waves have been obtained^31^. However, a systematic assessment on the predictability of rogue waves in terms of, for example, the forecasting horizon and accuracy, is not currently available.

In summary, it is unclear whether rogue waves are predictable at all, and hence, no rogue wave forecasting algorithm is available. Through this study, the authors aim to fill this gap by predicting the emergence of rogue waves from field data recorded with surface buoys. This represents an advancement in two major directions. First and foremost, from a practical perspective, these predictions pave a path to rogue wave forecasting systems that could be employed to enable safer offshore operations. Additionally, the sufficiency of current ocean wave measurements to predict rogue waves is investigated. Second, from a theoretical viewpoint, the presented investigations also shed light into the considerations about the predictability of rogue waves, and in turn, on the underlying rogue wave formation mechanisms.

**Figure 1:**
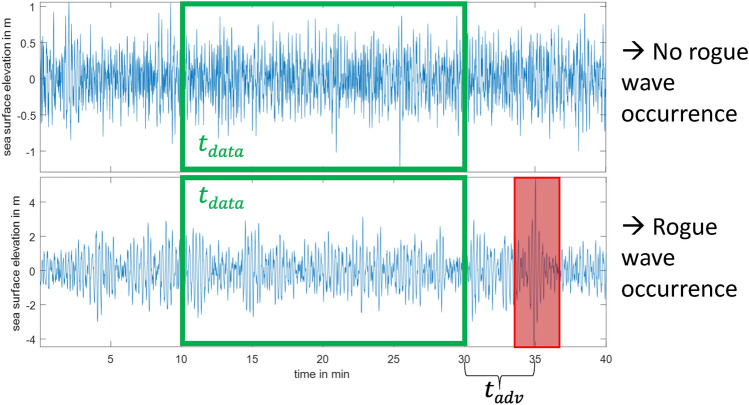
Two measurements of the sea surface elevation. While a rogue wave occurred in the time series shown in the bottom at about 35 min, no rogue wave emerges in the measurements shown in the top. Based on the recordings of length $t_{data}$ (i.e., *prior* to the rogue wave event for the measurements shown in the bottom), a rogue wave forecasting system decides whether a rogue wave with an advance warning time of $t_{adv}$ will occur or not.

The buoy measurements considered in this article are single point observations of the sea surface elevation as shown in Fig. 1. Rogue waves are predicted from such measurements as follows. Based on measurement taken over a duration of $t_{data}$ (cf. the green frame in Fig. 1), a rogue wave forecasting system needs to decide whether a rogue wave will occur within the duration $t_{adv}$ or not. For the measurement shown at the top of Fig. 1, the prediction should be ‘no rogue wave will occur’ while for the time series shown in the bottom, a rogue wave forecasting system should issue a warning. This understanding naturally gives rise to the following classification: i) ‘non-rogue-wave’ samples that are wave recordings after which no rogue wave occurs and ii) ‘rogue-wave’ samples that are sea surface measurements preceding a rogue wave. It is important to note that for meaningful advance warning times $t_{adv}>0$ selected in the following, the rogue wave itself is not included in the rogue-wave samples. Furthermore, the focus of this approach is solely on forecasting rogue wave occurrences within the advance warning time $t_{adv}$. Other characteristics, such as the height of the rogue wave, are not predicted, but could be obtained in future studies.

Fundamentally, if rogue waves are predicable, then there is a functional relationship between the waves that have occurred prior to the rogue wave event (i.e., inside the green frame in the bottom of Fig. 1) and the rogue wave event itself (cf. the red frame in the bottom of Fig. 1). If this functional relationship exists, it can be approximated with a universal function approximation. Thus, employing a universal function approximator can reveal if rogue waves are predictable.

In this work, neural networks, more specifically long short-term memory networks^32^, are utilized to predict rogue waves. With their universal function approximation capabilities^33^ these networks have, at least in principle, the capabilities to uncover any functional relationship to predict rogue waves, if such a functional relationship exists. This non-parametric model choice is also owed to the complexity of the oceanic environment, the sparsity of available buoy measurements, and the unclear formation mechanisms of rogue waves at sea, as it alleviates the necessity to rely on modelling assumptions. Furthermore, with the selection of neural networks, recent advances of non-parametric, model-agnostic, and data-driven modelling approaches in engineering and applied sciences are leveraged^34,35^.

## Methods

The buoy data^36^ is utilized to parameterize neural networks for rogue wave predictions as illustrated in Fig. 1. In the following, the authors provide an overview of the buoy data used. Subsequently, the extracted rogue waves, and the utilized neural networks are detailed.

### Buoy data

The data set is provided and maintained by the Coastal Data Information Program (CDIP), Scripps Institution of Oceanography^36^. In total, this database comprises measurements from 172 buoys. These buoys are either Datawell directional waveriders MkIII^37^ or Datawell directional waveriders MkIV^38^. Amongst other sensors, these surface buoys are equipped with accelerometers from which the buoy’s vertical displacement is deduced. After internal signal processing, for example, bandpass filtering, the buoy’s vertical displacements are sent ashore and stored. The sampling rates of the stored data are 1.28 Hz for the MkIII version and 2.56 Hz for the MkIV system. In general, these surface buoys closely follow the sea surface elevation and are commonly utilized to deduce the sea surface elevation. It is noted surface buoys have a tendency to avoid large wave crests through lateral movements^39^ and linearize the wave profiles^40,41^. Nevertheless, buoy measurements have been extensively validated^11,39,42^, and in conjunction with laser measurements, buoy measurements yield the most reliable and extensive rogue wave observations^7^.

**Figure 2:**
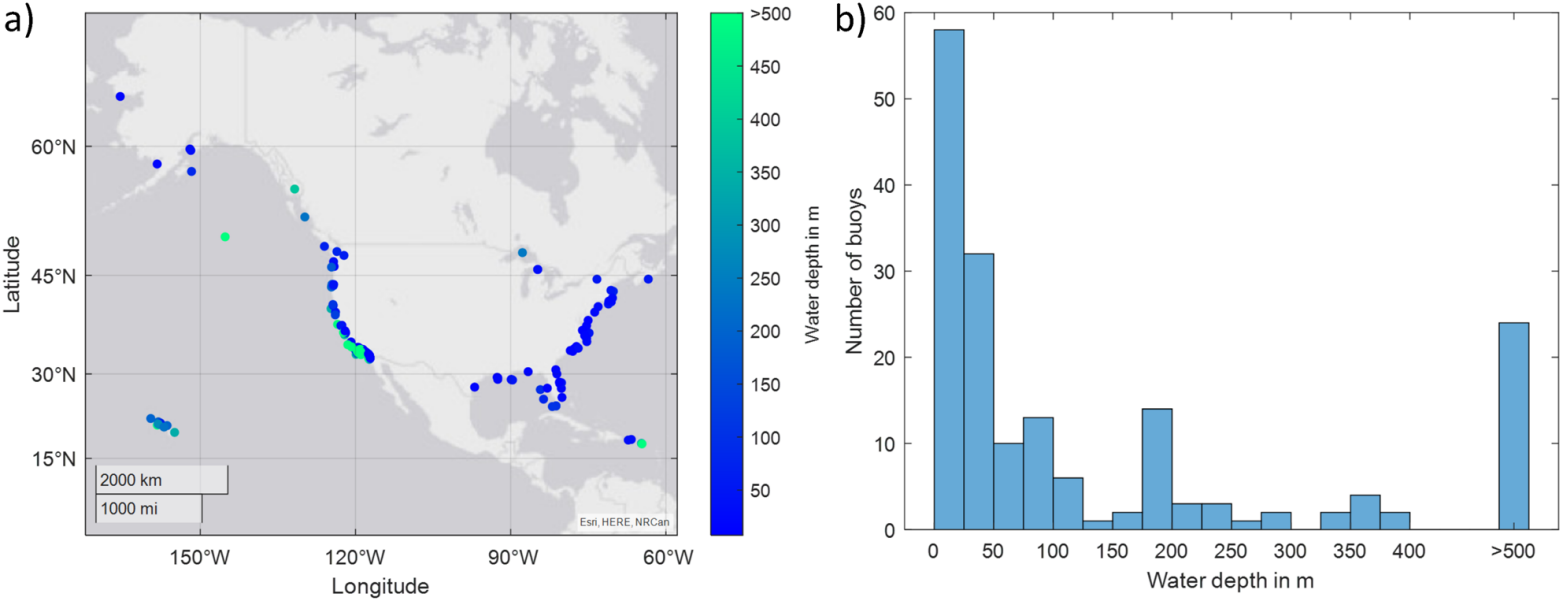
Buoy network^36^: (**a**) Location of the ocean buoys^36^. Additional buoy are located near Floripa (Brazil), Anuu (American Samoa), Saipan (US), Guam (US), Palau (Marshall Islands) and Majuro (Marshall Islands). The figure was generated using MATLAB^®^ (Version: R2021a) . (**b**) The mooring depth on the individual buoys shown in a histogram. The water depth varies from a few meters up to more than 4000 m.

The CDIP-buoys are primarily located near the shores of the continental US (cf. Fig. 2a) while some buoys are located near Pacific Islands. The buoys located in sounds and lakes are excluded in the following as this study focuses on the occurrences of oceanic rogue waves. More specifically, recordings from buoys with the CDIP identifier number 175, 177, 204, 205, 221, 230, 248, 251, and 253 are not considered. Waves measured at these sheltered locations differ noticeably from recordings from the open ocean. The water depths at which the ocean buoys are deployed vary considerably from a few meters to more than 4000 m (cf. Fig. 2b). While many buoys are located in shallow water, more than 20 buoy are deployed in deep water with a depth of more than 500 m. More information on the buoy network, including interactive maps and plotting tools can be found in^36^ and the accompanying websites.

The observation network covers a large area, and hence, buoys are usually separated by several kilometers. Since rogue waves are inherently localized in space and time, the spatial resolution is to coarse and irregular to trace the evolution of individual rogue waves. Thus, the space-time evolution of individual rogue waves cannot be followed in this data set. Hence, no spatial information on the rogue waves is kept and no predictions on the evolution of a rogue wave in space is made in the following. To realize such predictions, measurements with higher spatial resolution need to be considered.

The measurement starts and durations vary from buoy to buoy. In total more than 20 billion ($20\cdot 10^9$) samples of sea surface elevation are contained in the data set^36^. This sample size is equivalent to 16 million half hour intervals or 880 years of consecutive data. Before organizing the buoy measurements into data sets and identifying individual rogue waves, this vast data set is quality controlled. The employed quality control is detailed in the Supplementary Material S.1).

**Figure 3:**
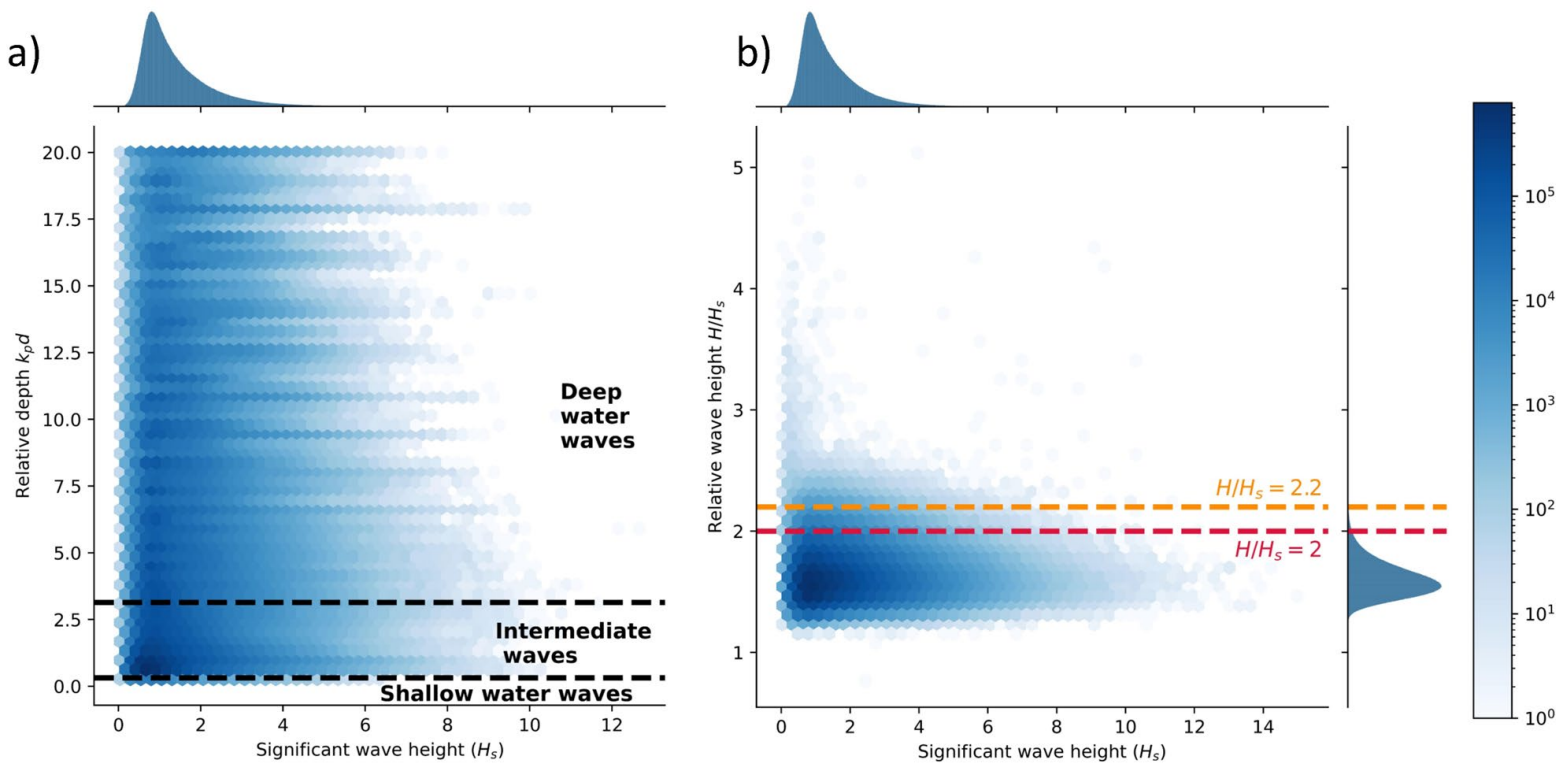
Overview over the extracted wave data^36^. The intensities are plotted in a logarithmic scale to emphasize the tails of the distributions: (**a**) significant wave height ($H_s$) versus relative depth defined as peak wave wave number ($k_p$) times deployment depth (*d*). (**b**) Significant wave height ($H_s$) versus relative wave height ($H/H_s$).

In Fig. 3 an overview over the quality controlled wave data is provided. Therein, the intensities are plotted on a logarithmic scale to emphasize the tails of the distributions. The significant wave height $H_s$ is calculated as four times of the standard deviation of the sea surface elevation. This significant wave height ranges from less than one meter to more than 10 m. Calculating the relative depth by multiplying the peak wave number $k_p$ with the deployment depth reveals that most wave measurements are classified as deep (55%) or intermediate water waves (45%). Only a marginal portion of shallow water waves is included (cf. Fig. 3a). Furthermore, the relative wave height $H/H_s$, defined as the quotient of the the maximal wave height *H* (from through to crest) within the half hour intervals and the significant wave height $H_s$, is between one and two for most measurements (cf. Fig. 3b). However, for some recordings larger relative wave heights $H/H_s$ are reported. More specifically, about 1.23% of the half-hour measurements contain a wave with a relative wave height larger than two ($H/H_s>2$) and only $0.14\%$ contain a wave with a relative wave height larger than 2.2 ($H/H_s>2.2$). Those samples are characterised as rogue waves in the following section.

### Rogue waves

After passing through the quality control (cf. Supplementary Material S.1), the 30-min long measurements are scanned for rogue waves. To this end, the significant wave height $H_s$ is calculated (four times of the standard deviation of the sea surface elevation), and the wave heights *H* (from through to crest) and the crest heights $\eta _c$ are extracted. Then, the following three rogue wave definitions

1a $$\begin{aligned}{} & {} \frac{H}{H_s}>2.2, \end{aligned}$$

1b $$\begin{aligned}{} & {} \frac{H}{H_s}>2, \end{aligned}$$

1c $$\begin{aligned}{} & {} \frac{\eta _c}{H_s}>1.25, \end{aligned}$$

from the literature (cf., e.g.,^4,7,21,22,25,30,43^) are considered. If one of the definitions (1) is satisfied, then the corresponding sea surface measurement is normalized by the significant wave height and stored. Therein, each definition is treated separately, and this yields three different collections of rogue waves. Before storing, each time series is shifted so that the rogue wave occurs at minute twenty-five. Since this shifting could corrupt the extracted rogue waves with measurements that did not pass the quality control, the shifted time series is quality controlled again (cf. Supplementary Material S.1). As a final check, ten percent of all saved rogue wave samples are randomly selected for visual inspection and no irregularities are detected. Admittedly, the authors have iteratively designed the quality control in Supplementary Material S1 such that only physical rogue waves remain in the final data sets. More specifically, starting with only the first two quality flags yields rogue waves data sets with the irregularities shown in Supplementary Figure S.1. Subsequently, imposing the three additional quality flags, based on physical insights, removes all unrealistic rogue wave recordings.

Along with the rogue wave samples, sea surface measurements without rogue wave events are stored. In Supplementary Table S.1 (cf. Supplementary Material S.3) the authors provide a detailed overview over the extracted rogue waves. For each rogue wave sample, a time series without a rogue wave from the same buoy is randomly selected and stored. Thereby, control times series are obtained, which differ from stored rogue wave samples only in the aspect that they do not include a rogue wave. The obtained data sets are summarized in Table 1. This procedure yields data sets with an equal number of samples with and without rogue waves. For future applications, the ratio between rogue-wave samples and non-rogue-wave samples could be varied. Due to the large sample sizes and the random selection of the control time series, it expected that those yield a good representation of commonly occurring sea states in the ocean.

**Table 1:** Overview of the prepared data sets.

Data set	Rogue wave definition	Number of samples with rogue waves	Number of samples without rogue wave
A	(1a)	$~19\cdot 10^3$	$~19\cdot 10^3$
B	(1b)	$172\cdot 10^3$	$172\cdot 10^3$
C	(1c)	$~27\cdot 10^3$	$~27\cdot 10^3$

Comparing definitions (1a) and (1b) reveals that the rogue wave contained in the data set A are also part of the data set B. This is not necessarily true for the data set C, which contains rogue waves with large crest heights, rather than large wave heights as the data sets A and B. However, a large overlap between the data set C with the other two data sets is expected.

### Neural networks

Traditionally, neural networks have been employed, for example, image recognition^44^, language translation^45^, and speech recognition^46^. More recently, the use of these networks has shown promising progress for challenging problems, such as protein folding^47^, global weather forecasting^48^, and large language modelling^49^. Deep neural networks hold the promise for approximating any functional relationship between input data and output data^33^, if enough data for parameter tuning is available and if the envisioned functional relationship exists. Hence, at least in principle, those can be utilized to forecast rogue waves. If successful not only a very practical rogue wave forecasting system can be obtained but also the predictability of rogue waves can be quantified by using field measurements.

Recurrent neural networks with long short-term memory (LSTM)^32^ are employed within this work. Initial attempts with alternative architectures, more specifically convolutional neural networks and transformer networks, yielded results inferior to the results obtained with LSTM-networks. Exploring alternative network architectures will remain an important direction for future research. Recurrent neural network have been developed for tasks with sequential data and feature hidden, internal states, which can be used to store the temporal history of the data^35,46^. As a distinction, LSTM-networks are designed to flexibly erase and retrain their internal states. Such networks have been utilized to obtain data-driven forecasts of complex systems, such as high-dimensional, chaotic systems^50^, extreme events^51^, and ocean waves^31^.

From each sample, a recording with the duration $t_{data}$ is extracted. For the rogue-wave samples, this recording ends $t_{adv}$ prior to the rogue wave event, yielding the advance warning time $t_{adv}$ (cf. Fig. 1). In this setting, the neural network is utilized to distinguish between time series before a rogue wave event and measurements which do not precede a rogue wave. This approach differs from common forecasting approaches^31,50,51^ and the forecasting problem has been rephrased into a time series classification task. This shift is motivated, first, by the high maturity of neural network architectures in classification tasks, while applications to time series data is comparably still less common. Moreover, from a practical perspective, it is of foremost interest to know if a rogue wave occurs or not. Information about the height of the rogue wave or other intermediate sea surface elevations are of secondary importance. Future studies could increase the forecasting content to, for example, also generating a prediction for the rogue wave height.

**Figure 4:**
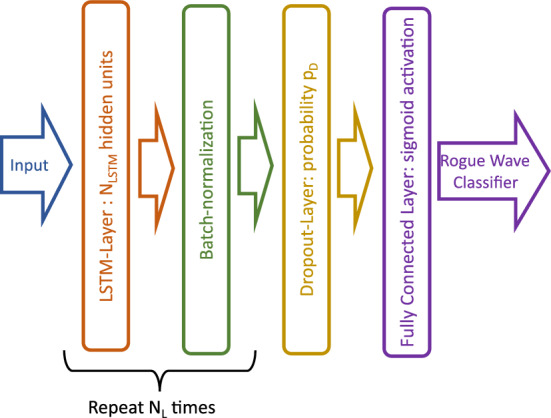
Architecture of the utilized LSTM-network.

The utilized neural network architecture is illustrated in Fig. 4. First, $N_L$ LSTM-layers alternate with layers performing batch-normalization. Each LSTM-layer consists of $N_{LSTM}$ hidden units arranged in parallel. The batch-normalization layers scale each state to have zero mean and unit variance. These layers are followed by a dropout layer that is used to set every feature to zero with a probability of $p_D$. The dropout layer have been designed to avoid overfitting^52^, which is a common issue for neural networks with many parameters^53^. As a final layer, a fully connected layer is used at the end of the network. This layer is used to reduce the feature size to the number of output classes (i.e., two in this paper). Moreover, a nonlinear, sigmoid activation function is included.

The output of the network is a two-dimensional probabilistic classifier with the probabilities $p_{RW}$ and $p_{NRW}$. These probabilities indicate the likelihood with which the supplied sample belongs to one of the corresponding classes; that is, non-rogue-wave sample and rogue-wave sample. To yield a definitive prediction, the class with the higher probability is selected as the forecast.

Following the common procedure, the data sets are randomly split into training (64 %), validation (16 %), and testing (20 %) data. The testing data is reserved to evaluate the final performance of the neural network. For parameter tuning, only the training and validation data are used with the network. The weights of the neural networks are obtained via the stochastic gradient implemented within the Adam optimizer^54^. The learning rate is scheduled following the cosine-decay with an initial learning rate of 0.001. The final hyperparameter choice is reported in Table 2.

**Table 2:** Values of the hyperparameter.

Parameter	Values
Number of hidden states $N_{LSTM}$	10
Number of stacked Layer $N_L$	4
Dropout probability $p_D$	0.05
Batchsize	32

With the hyperparameter choice given in Table 2, the network has 3182 trainable parameters. This number is relatively small compared to employed neural networks for other applications. The hyperparameter selection has been compared to results of state-of-the-art hyperparameter tuning algorithms^55^ and self-programmed random grid searches. Those approaches can only sometimes increase the performance further, indicating that the simple choice reported in Table 2 is near optimal. Selecting these values also allows the authors to utilize a single network architecture in all experiments. More extensive parameter tuning studies could be an appealing future approach to increase the forecasting accuracy.

The networks are constructed by using Tensorflow^56^ (version 2.9.1) and the training is conducted either by utilizing a local NVIDIA Quadro P1000 GPU unit or a NVIDIA A100 GPU unit made available via the University of Maryland supercomputing resources.

## Results

In the following, results of rogue wave forecasting are presented. In all cases, 20 min of measurements are made available to the neural network ($t_{data}=20$ min in Fig. 1), while the advance warning time is varied. First, perfectly balanced datasets containing an equal number of samples prior to a rogue wave and samples without a rogue wave are utilized to train neural networks for rogue wave forecasting. Subsequently, the extrapolation capabilities of the trained neural network are verified. Finally, the real ocean is emulated by considering a heavily imbalanced dataset, which contains a surplus of non-rogue-wave samples and only very few samples preceding a rogue wave.

### Balanced data sets

**Figure 5:**
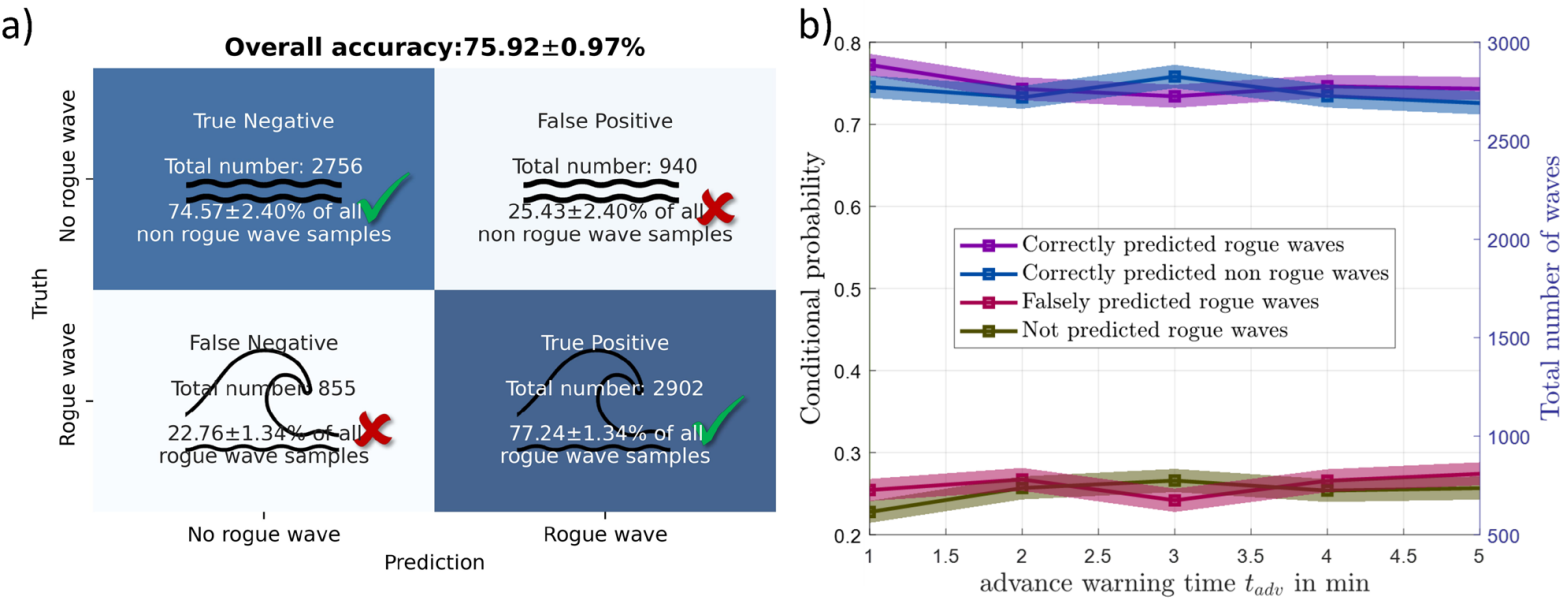
Predictions from the LSTM-network for testing portion of the data set A ($$H/H_s>2.2)$$: (**a**) Confusion matrix with 95% confidence intervals for an advance warning time $$t_{adv}$$ of 1 min. (**b**) Variations of the prediction accuracies with respect to advance warning time. The shaded regions demarcate 95% confidence intervals.

First, the data set A consisting of about 40 thousand samples (cf. Table 1) is considered. Selecting an advance warning time $t_{adv}$ of 1 min yields the result shown in Fig. 5a. The prediction of the neural network can be that either a ‘rogue wave will happen’ or a ‘no extreme wave will occur’ and the combination with the the truth; that is, either ‘rogue wave occurred’ or ‘no extreme wave observed’, yields the following four combinations: ‘True positive’, ‘True negative’, ‘False positive’, and ‘False negative’.

About 3000 rogue waves are correctly predicted by the neural network (cf. ‘True positive’ in Fig. 5a), which is about 77% out of all rogue-wave samples (2902 out of 3757). Hence, about three out of four rogue waves are correctly forecast 1 min in advance. Similarly for about 2800 samples no-rogue wave warning is issued from the neural network, which amounts to 75 percent of all non-rogue-wave samples. The two fields ‘False negative’ and ‘False positive’ summarize the wrong predictions from the neural network. In total, the network misses to alert for 855 rogue waves or equivalently about 23 percent. Similarly, in one out of four cases, network predicts an upcoming rogue wave although no such wave occurred in reality (cf. ‘False positive’ in Fig. 5a)

The total accuracy (percentage of correct predictions) of the trained neural network on the testing data set is 76 percent or equivalently three out of four predictions of the neural network are correct. The 95% confidence interval of this binomial distribution^57^ is estimated to be less than 1%. About 3000 rogue waves have been correctly predicted. To the best of the authors’ knowledge, this is the most extensive rogue wave prediction experiment that has been carried out with field data.

**Figure 6:**
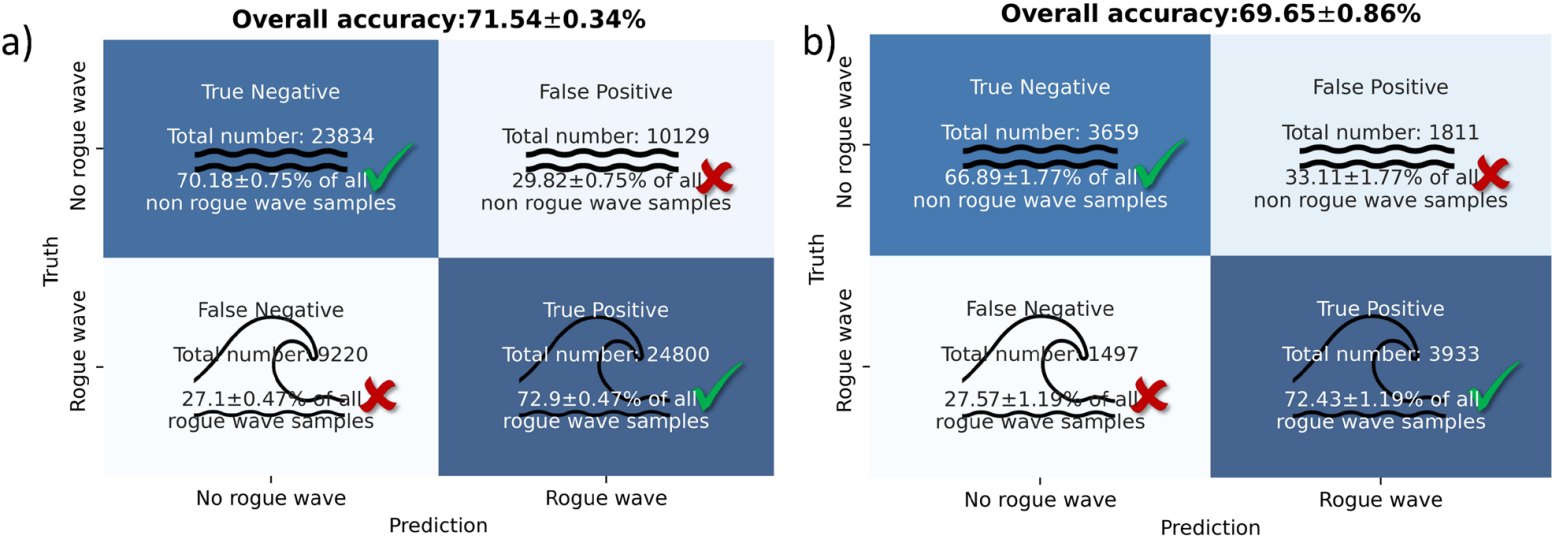
Predictions with 95% confidence intervals from the LSTM-network for testing data with an advance warning time $$t_{adv}$$ of 1 min: (**a**) Data set B ($$H/H_s>2)$$. (**b**) Data set C ($$\eta /H_s>1.25$$).

During the advance warning time safety can be enforced by, for example, seeking shelter, performing an emergency shutdown, or maneuvering, to minimize the impacts of an approaching rogue wave. Hence, it is of practical importance to maximize the advance warning time. Therefore, the advance warning time is increased in 1-min increments. For each advance warning, time a neural network is retrained and its performance is evaluated. Selecting $t_{data}=20$ min yields the maximal advance warning time of 5 min with the compiled data set, since 25 min of sea surface elevation prior to every rogue wave are stored in the data sets (cf. Section “Rogue waves”). The results of these experiments are included in Fig. 5b. Therein, it is discernible that the number of correct predictions remains high for all advance warning times while a decrease of the forecasting accuracy from 76 percent to 73 percent is noted. Generally, a declining accuracy with increased advance warning time is expected, as one expects a lower correlation between waves separated further in time. However, as shown in Fig. 6, this decline is rather gradual. This slow decay of the forecasting accuracy indicates that rogue wave predictions with advance warning time of multiple minutes are within the realm of possibility.

After utilizing the data set A for the forecasting experiments, the data sets corresponding to the two alternative rogue wave definitions (1b) and (1c) are investigated. Selecting an advance warning time $t_{adv}$ of 1 min yields the results shown in Fig. 6. For both data sets, a good portion of the upcoming rogue wave is correctly predicted: in total about 25.000 rogue waves for data set B (cf. ‘True Positive’ in Fig. 6a) and approximately 4000 rogue waves for data set C (cf. ‘True Positive’ in Fig. 6b). Moreover, 70% of the non-rogue-wave samples are correctly detected for the data set B, and similarly, 67% for data set C. The percentage of correct predictions is 72% for data set B and 69%for data set C.

The forecast accuracy for both data sets B and C (cf. Fig. 6) is less than the accuracy obtained for data set A (cf. Fig. 5a). More specifically, a difference of 4 percent between the accuracy for data set A and accuracy for data set B is observed (76 percent for data set A compared to 72 for data set B). Data set B contains data set A and is about ten times larger than data set A (cf. Table 1). These observations allow for the following conclusion. First, due to the larger size of data set B, it is expected that a network with a higher number of parameters is necessary to capture an underlying function distinguishing rogue-wave samples from non-rogue-wave samples. Hence, one can explain the decreased performance with the network architecture (cf. Fig. 4) and hyperparameter choice (cf. Table 2). Furthermore, one could expect that the larger data set B would allow one to tune a more powerful network architecture with a higher number parameters. However, preliminary hyperparameter tuning with the architecture shown in Fig. 4 was not found to yield a significant performance increase. On the other hand, more data does not necessarily imply that a higher accuracy is achievable. Indeed, the additional samples included in data set B could diminish the differences between the two classes, and hence, could make forecasting more challenging. The presented forecasting experiments seem to suggest that a less strict rogue wave definition (compare definition (1b) and (1a)) impedes correct rogue wave forecasts. Indeed, the larger threshold in the definition for the data set A yields more extreme rogue waves (i.e., further in the tail of the wave distribution) compared the lower threshold for data set B. Thus, one can hypothesize that those more outstanding samples share common patterns or characteristics, which makes them easier to predict.

The accuracy for the data set C is less than that for the data sets A and B. Data set C is about two times larger than data set A, and five times smaller than data set B. A distinction of data set C is that the rogue wave definition relies on the crest height $\eta _c$ compared to the wave height *H* (from crest to trough) for data sets A and B. Thus, data set is not necessarily a part of data set B (or A), although a large overlap is expected. From a purely data-driven perspective, one can conclude that reliance on the crest height makes rogue waves more difficult to predict. It is especially notable, that the percentage of detected rogue waves is comparable to that obtained for data set B, while a comparably high percentage of false alarms are raised (cf. ‘False Positive’ in Fig. 6b). In the experiments conducted in this work, it is found that the neural network tends to overpredict the likelihood of rogue wave occurrences.

### Extrapolation—zero-shot experiment

After successfully predicting thousands of rogue waves, the following question arises: How valuable or universal are the trained neural networks for rogue wave forecasting? In practise rogue wave forecasts should not be restricted to the measurement locations included in the training data, but also be valid for other locations. This requires one to evaluate the trained network for locations not included in the training data (i.e. zero-shot learning^58^). Now, if the rogue wave forecasting function approximated by the trained networks is truly universal, then this approximation should also carry over to locations not contained in the training data. In general, as neural networks are poor extrapolators, it is not self-evident whether the rogue wave forecasts obtained in the preceding section are useful for any locations other than the buoy locations contained in the training data (cf. Fig. 2). However, if the training data is extensive enough and comprises all typical sea states, then data stemming from a new buoy location could be similar enough to the training data such that the neural network’s predictions are accurate for the new buoy location as well. In this case, the trained neural network would indeed be a universal rogue wave predictor. The prepared buoy data^36^ consists of thousands of rogue waves (cf. Table 1) and millions of non-rogue waves, which, in principle, could enable universal rogue wave forecasts.

**Figure 7:**
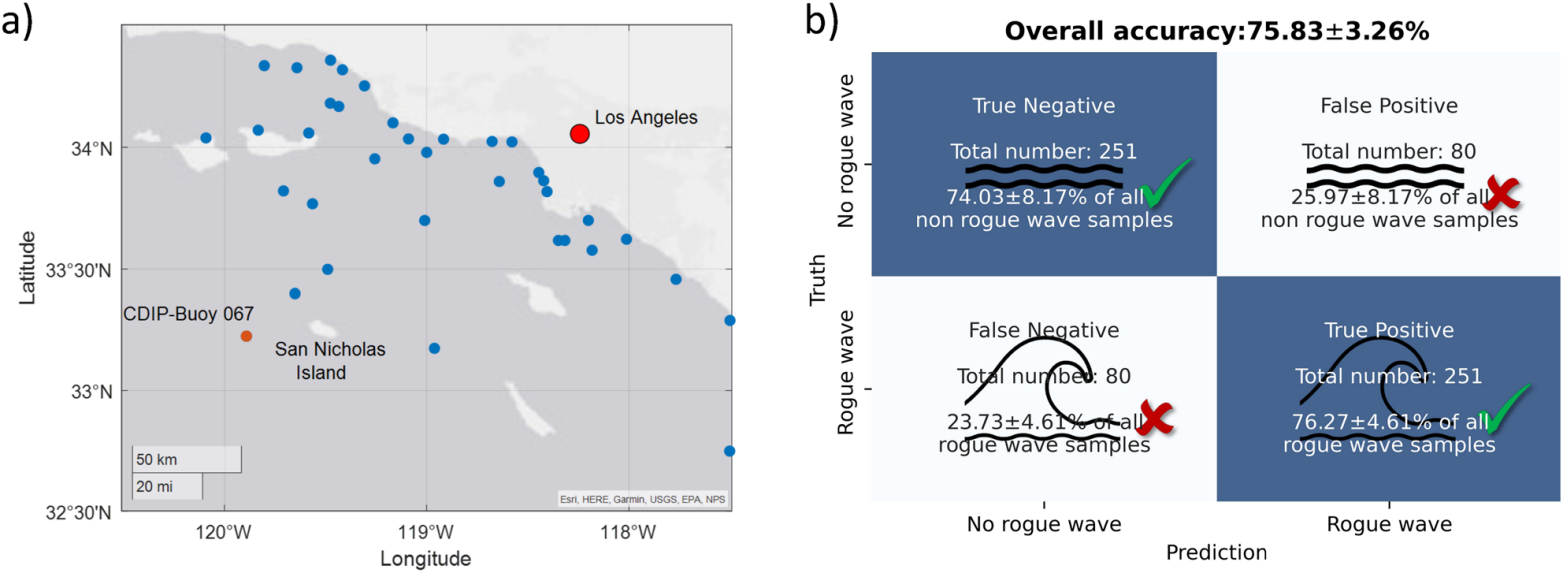
Extrapolation of the LSTM network to Buoy 067: (**a**) Location of the CDIP Buoy 067. The Figure was generated using MATLAB^®^ (Version: R2021a). (**b**) Predictions with 95% confidence intervals from the neural network for the Buoy 067.

To test the universality of the neural networks, the forecasting experiments presented in the preceding section are repeated while excluding one buoy from the data set. More specifically, all measurements from CDIP Buoy 067 are removed from the data set A. Then, a neural network is retrained on the remaining buoy data (excluding Buoy 067), and rogue wave predictions are made for the measurements from Buoy 067. Buoy 067 is located near San Nicholas Island off the coast of Los Angeles (cf. Fig. 7a). The water depth at this location is 315 m. In total, 331 rogue waves with a wave height exceeding the significant wave height by a factor of 2.2 ($H/H_s>2.2$) were detected at this location. These rogue waves and corresponding non-rogue-wave samples are excluded from the training data utilized to parameterize the neural network. The distance from the location of Buoy 067 to the next nearest buoy is about 30 km. Due to this large separation, the individual waves measured with Buoy 067 differ from the recordings of the other buoys in the network. Hence, no knowledge of the specific sea surface elevation at this measurement location is available to the neural network while training.

For an advance warning time $t_{adv}$ of 1 min, the neural network is found to show the same performance on the remaining buoy data as discussed in the preceding Section “Balanced data sets”. Now, evaluating the neural network on the measurements from Buoy 067 yields the forecasts shown in Fig. 7b. In total, 251 out of the 331 rogue waves are correctly predicted by the neural network. The same number of correct predictions is obtained for the non-rogue-wave samples. The percentage of accurate predictions is comparable to the performance on balanced data set (cf. Fig. 5a).

To further investigate the network’s extrapolation capabilities, the zero-shot experiment is repeated for two additional buoys. For a second trial, Buoy 132, located on the eastern shore of US close to Jacksonville, Florida, is selected. The deployment depth is 15 m that is significantly more shallow than the water depth for Buoy 067 (315 m). The distance from Buoy 132 to the nearest buoy included in the data set is about 80 km. As a final trail, Buoy 166 is selected. This buoy has not only the deepest deployment depth of the data set (4254 m), but is also extremely remotely located in the Pacific. The nearest buoy included in the data set is more than 900 km away from Buoy 166. For both experiments, the shallow water Buoy 132 and the extremely remote Buoy 166 in deep water, the network’s forecasting accuracy is 75% (cf. Supplementary Fig. S.2 in Supplementary Material S.2).

In summary, the extrapolation experiments for all three locations; that is, deep water buoy at the US west coast, shallow water buoy at the US east coast, and a remote buoy in the Pacific, yield a comparable accuracy to the forecast accuracy when measurements from these locations are included in the training data. This exemplifies the extrapolation capabilities of the trained neural network. Based on the conducted experiment, the approximated functional relationship between waves preceding a rogue wave and the rogue wave event does indeed seem universal.

### Unbalanced data sets—employment in the real ocean

In the two preceding sections, the performance of neural networks to forecast emerging rogue waves is demonstrated with perfectly balanced data sets containing an equal number of rogue-wave and non-rogue-wave samples. However, in reality, rogue waves are rare and non-rogue-wave samples prevail. This observation stimulates the following question: How do neural network approaches perform in a more realistic setting when non-rogue wave samples are dominant in the data collected? To answer this question, an unbalanced data set emulating realistic conditions at sea is considered.

The neural network for rogue wave prediction can be trained offline before employment of the system. Hence, during training, the ratio between rogue-wave and non-rogue-wave samples can be arbitrarily controlled and it does not necessarily need to represent the ratio observed in the real ocean. In the following, the training data is selected to be perfectly balanced, which allows to utilize the neural network trained in the Section “Balanced data sets”. In general, the ratio that maximizes the forecasting system’s performance would be most favorable. This optimization is remains an important aspect to explore in future studies. Once employed, the ratio is determined by the condition at sea and cannot be arbitrarily controlled. Therefore, the testing data for the neural network needs to contain a vast majority of non-rogue wave samples to emulate the real ocean.

**Figure 8:**
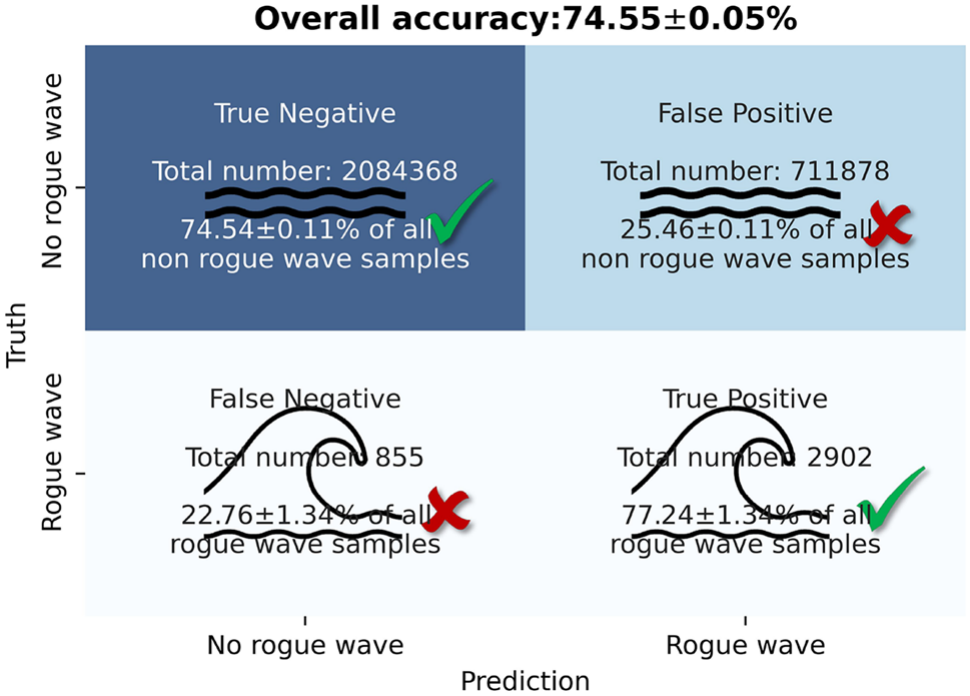
Predictions and confidence intervals from the LSTM-network for unbalanced testing data with a realistic ratio of rogue-wave samples to non-rogue-wave samples.

To emulate realistic conditions at sea, the testing portion of data set A is enriched by 2.8 million non-rogue-wave samples. This number corresponds to twenty percent of all 14 million quality controlled 30-min long time windows obtained from the buoy data^36^. Thereby, only 0.14 percent of the samples in the testing data are rogue-wave samples (equal to 3800 rogue-wave samples). After evaluating the trained neural network for an advance warning time $t_{adv}$ of 1 min on the enriched testing data set, the result shown in Fig. 8 is obtained. Therein, the number of correctly predicted and missed rogue waves is the same as in Fig. 5a. This is to be expected since the rogue wave samples and trained neural network are the same.

For the non-rogue-wave samples, the neural network is found to overwhelmingly correctly predict no upcoming rogue waves. The percentage of ‘True Negatives’ is similar to that seen in Fig. 5a, while the absolute number is significantly higher. This indicates that the performance of the neural networks trained with balanced data sets carries over to realistic conditions with an overwhelming majority of non-rogue-wave samples. Hence, the neural networks trained in the Section “Balanced data sets” can be readily employed in realistic condition without sacrificing accuracy.

From Fig. 8, one can also discern that for such unbalanced data sets the total prediction accuracy, defined as the number of correct predictions divided by the number of all predictions, is not a good performance metric to maximize. The total accuracy from Fig. 8 is about 75% . This performance is easily exceeded by the trivial prediction ‘no rogue wave will occur’ for all samples, which yields a total accuracy of 99.86%. However, this prediction does not capture a single rogue wave, and hence, this is not a useful rogue wave predictor. For the balanced data sets discussed in the Sections “Balanced data sets” and “Extrapolation—zero-shot experiment”, this issue does not arise.

## Discussion

In the preceding section, thousands of rogue waves are predicted from buoy data. More specifically, neural networks were found to be able to predict three out of four rogue waves minutes in advance. The trained neural networks are found to extrapolate well to new buoy locations, indicating the universality of the approximated forecasting function. Moreover, the performance of the neural networks is found to carry over to realistic conditions with an abundance of non-rogue-wave samples. These encouraging results deserve further discussion.

The correct predictions of thousands of rogue waves from measurement are unprecedented in the literature, and as such, demarcates a significant step towards reliable rogue wave forecasting. However, the fact that about three out of four rogue waves are predicted also implies that one out of four rogue waves is not predicted and that a significant number of false alarms are issued (cf. Fig. 8). For an operational system, this accuracy has to be increased further. To this end, the data-driven approach taken can be altered by employing more powerful neural network architectures. For example, transformers with multi-head attention^59^ or encoder-decoder networks^60^ could be utilized. However, it needs to be noted that these network architectures come with a higher number of parameters, and usually more data is necessary for training. To this end, de-spiking algorithms (e.g.^64^) could be employed to remove spikes detected in the quality control rather then discarding the corresponding measurement (cf. Supplementary Material S1). This could increase the number of detected rogue waves. If the growing data set^36^ does not suffice, then alternative ocean wave measurements need to be obtained.

Moreover, the fact that three out of four rogue waves are predicted sheds light on the answer to the following basic question: Is the occurrence of a rogue wave predictable? Based on the current work, one can state that some rogue waves are predictable but this does not conclusively rule out the theory of superposition of elementary waves with random phases^7^ implying unpredictability of rogue waves. Indeed, theoretically, a portion of the missed rogue waves could be generated by this mechanism. To further investigate the generation mechanisms of rogue waves, one could dissect the rogue wave data set (cf. Table 1) into rogue waves that seem predictable and the remaining extreme waves which are not predicted by the neural network. A subsequent analysis could reveal fundamental differences between the two postulated rogue wave types and help to refine the rogue wave definitions as pointed out in reference^21^.

The data-driven approach presented draws its power from its model agnostic generality and the universal function approximation capabilities of the utilized neural network. Besides rogue wave predictions, no direct physical insights are gained, at first. However, future studies could lead to more physical insights. For example, information about the water depth, wind speed, or buoy location could be supplied to the neural network and the impacts of these parameters on the forecasting accuracy can be observed. Especially, supplying wind speed seems a promising direction, as it is generally acknowledged that wind is a key factor for the generation of ocean waves^12^. Moreover, tools and procedures from explainable or interpretable artificial intelligence could be utilized to gain further insights into the forecasting function approximated by the neural network. Indeed, decision trees^61^, saliency methods^62^, or integrated gradients^63^ have been used to explain deep neural networks for time series classifications. An application of these methods to the trained neural networks could reveal additional insights into the physical mechanisms of rogue wave formations.

The buoy data utilized in this study are single point observations and it is quite remarkable that with such limited information accurate forecasts are possible. Since waves travel in space, it is expected that a rogue wave predicted for a certain location will also be observed for other locations in the direction of travel. Hence, incorporating spatial observations of sea surface elevation into this data-driven approach would most likely significantly increase the forecasting accuracy as well as the advance warning time. Moreover, one could also capture application scenarios, where a warning from a sensor (buoy or optical measurement system) could be issued for a location (i.e., a ship or offshore platform) located nearby. Thus, enriching the presented approach by incorporating spatial information is an appealing future direction of research. To this end, a large number of quality-controlled sea surface measurements with a high-resolution in space and time are required. However, due to the difficulties measuring ocean wave at sea^11^ quality controlled and highly sampled measurements of ocean waves remain limited.

In the work presented, the neural networks have been trained to answer the following fundamental question: Will a rogue wave occur in $t_{adv}$ minutes? The question is simplified as much as possible to increase the forecasting success of the neural network. Therein, the underlying assumption is that a simpler question implies a simpler function for the neural network to approximate. If one were to obtain information about the height as well as impact time of the upcoming rogue wave, that information would also be of relevance in practice. To this end, one could utilize the data prepared in this article to forecast the heights of upcoming rogue waves. Moreover, one could also prepare the data sets to predict the time when a rogue wave occurs. While these ideas can spur promising future directions, it needs to be pointed out that more information that is desired will only make forecasts more challenging in all likelihood. Undoubtedly, more data will be required for parameter tuning of more powerful neural networks.

## Concluding remarks

In this study, impending freak or rogue waves are predicted from buoy data. The publicly available buoy data^36^ is scanned for rogue waves and thousands of 30-min long windows containing a rogue wave are extracted. Subsequently, ocean waves prior to the rogue wave event are extracted. These measurements are paired with recordings of equal length without rogue waves. Subsequently, an LSTM-network is utilized to distinguish between the two classes, namely, (i) waves preceding a rogue wave and (ii) waves not immediately followed by a rogue wave. This network is then used to predict rogue waves.

For an advance warning time of 1 min, three out of four (= 75%) rogue waves with a wave height exceeding the significant wave height by a factor of 2.2 ($H/H_s>2.2$) are predicted with the considered neural network. With an increase in the advance forecasting time, the accuracy of the rogue wave forecast is found to decrease. For example, for an advance forecasting time of 5 min, the neural network predicts only 73% of the supplied rogue waves. Similarly, altering the rogue wave definition by lowering the threshold ($H/H_s>2$) or considering the crest height $\eta _c$ ($\eta _c/H_s>1.25$) is found to slightly lower the forecast accuracy.

Additionally, the extrapolation capabilities of the trained neural network are tested, and it is demonstrated that the neural network extrapolates well to new buoy data. Indeed, withholding all measurements of specific buoys (i) deep water buoy of the coast of Los Angeles, (ii) shallow water buoy of the east coast of Florida, and (iii) a remote buoy in the Pacific) and subsequently testing the network’s performance on the withheld data also yields an accuracy of about 75% . This suggests that the trained neural network can serve as a rogue wave warning system for alternate locations. Moreover, this also indicates the universality of the approximated rogue wave prediction function. Finally, it is demonstrated that the performance of the neural network trained on balanced data sets with an equal number of rogue-wave samples and non-rogue-wave samples carries over to a real ocean, where a much higher percentage of non-rogue-wave samples is observed.

As discussed, future studies could improve the accuracy and advance warning time of this data-driven approach by, for example, by employing more powerful neural networks, supplying more physical information, or incorporating spatial wave measurements. To this end, more data will most likely be necessary to parameterize networks with a higher number of parameters and new sources with higher spatial resolution are required. It is expected that such approaches can improve the prediction accuracies obtained in this work.

Furthermore, despite the black-box character of the presented data-driven approach, physical insights can be gained. Within this work, it is clarified that rogue waves are to a large extent predicable with an advance warning time of a few minutes. Moreover, by systematically supplying or withholding information for the neural network, the importance of physical parameters for rogue wave predictions could be explored further. In addition, tools from explainable artificial intelligence could be used to study the parameterized networks in depth. Therein, it will be beneficial that the trained network consists of only about 3200 trainable parameters, which is a relatively low number compared to many state-of-the-art neural networks. Given that a freak wave or a rogue wave in the ocean is an example of extreme event, it is conceivable that the findings from the present work could also be utilized for predicting the occurrence of other extreme events in, for example, combustion processes^65^, seismic activity^66^ and possibly, climate^67^ based on observations.

### Supplementary Information

Supplementary Information.

## Data Availability

The ocean buoy data is publicly available^36^. All scripts, including the trained neural networks are publicly available at https://github.com/tbreunung/Freak-wave-forecasting. The data sets compiled and prepared are available at ^69^.
