# Supplementary material for: Prediction of freak waves from buoy measurements
Source: Sci Rep. 2024 Jul 18;14:16048. doi: 10.1038/s41598-024-66315-3 (PMC11258124; doi:10.1038/s41598-024-66315-3)
Supplement: Supplementary file 1 — Supplementary Information. [file 41598_2024_66315_MOESM1_ESM.pdf]

Supplementary material

Prediction of Freak Waves from Buoy Measurements

Thomas Breunung and Balakumar Balachandran
Department of Mechanical Engineering, University of Maryland, College Park, Maryland 20742, USA

S.1 Quality control of the buoy data [36]

Accurate measurements of waves in the open ocean remain challenging due to the harsh environment and inaccessible locations. Indeed, the vast data set [36] is corrupted by sensor failures, transmission errors, and other malfunctions, and hence, a strict quality control procedure is inevitable [30]. Similar observations have also been made for radar measurements of ocean waves [23]. To sort out unreliable data, the following series of steps are adapted.

First, the ocean wave measurements are divided into thirty-minutes long intervals. Over this duration, the statistics of the sea surface elevation is generally assumed as stationary and average sea state parameters can be calculated [25, 30, 39]. Then, for each window, the following quality criteria are checked:

1. Manufacturer quality flag: Datawell's waverider buoys are equipped with an automated quality control protocol for flagging unreliable data. If a single data point within the thirty-minutes long window is labelled as questionable, then the whole measurement is discarded.
2. Operator quality flag: CDIP personnel also regularly check the buoy data. To this end, the computed wave spectra and sea state parameters are inspected and faulty values are flagged. However, the raw time series utilized within this manuscript are not screened. To still benefit from the operator's expertise, this quality flag is utilized by discarding the whole thirty-minutes long time series, if a single sample is included in a wave spectra that has been flagged as unreliable by the CDIP personnel.
3. Spike detection: Moreover, the buoy data set contains measurements with unrealistic spikes as shown in Fig. S.1a. To sort out these spikes, the rate of change of the sea surface elevation is monitored and the threshold
$$S = \frac{4\pi\sigma}{T_z}\sqrt{2\ln N_z} \tag{S.1}$$
from reference [23] is utilized, wherein σ denotes the standard deviation of the sea surface elevation, T_z is the mean zero up-crossing period, and N_z is the number of zero up-crossings. If the absolute value of the rate of change surpasses the threshold value (S.1) once, then the whole time series is discarded. Therein, the rate of change is approximated with the first order finite difference; that is, by calculating the difference between two consecutive points and dividing by the sample period.
4. Sensor range: Additionally, wave crests exceeding the sensor range were found in the buoy data set. The measurements shown in Fig. S.1b seem to depict a large wave. However, the maximal wave crests of this recording exceeds 20 meters and is outside of the sensor range. Thus, this data is questionable. In the following, measurement windows containing a single wave crest height or through depth exceeding the sensor range are discarded.
5. Repeated values: Furthermore, in some measurements exactly the same value for the sea surface elevation is reported for multiple consecutive points (cf. Fig. S.1c). Considering the dynamic maritime environment and the ever-changing sea surface, this perfectly calm condition seems unrealistic. Hence, following earlier work [23], measurements that have the same sea surface elevation value for more than ten consecutive points are discarded.

Adapting the described quality control procedure leads to discarding of about ten percent of the buoy data [36]. More specifically, 14 million quality-controlled, thirty-minute windows of sea surface elevation measurements are obtained.

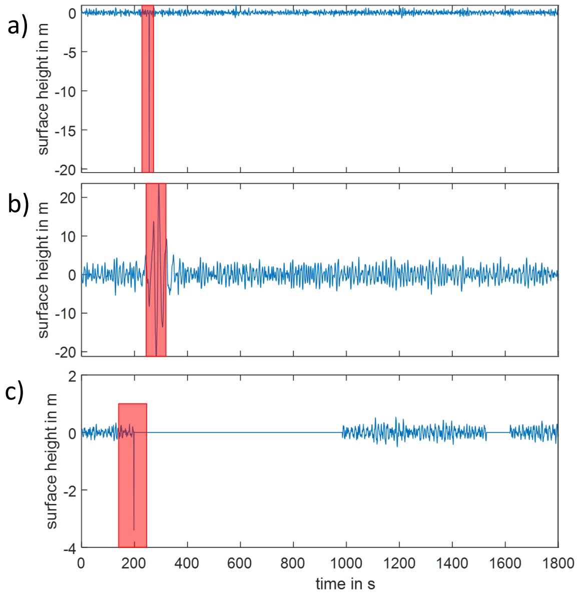

Figure S.1: Unreliable measurements contained in the buoy data [36]. a) Unrealistic spike. b) Sea surface elevation outside the sensor range. c) Consecutive data points with equal value.

S.2 Additional Extrapolation Experiments

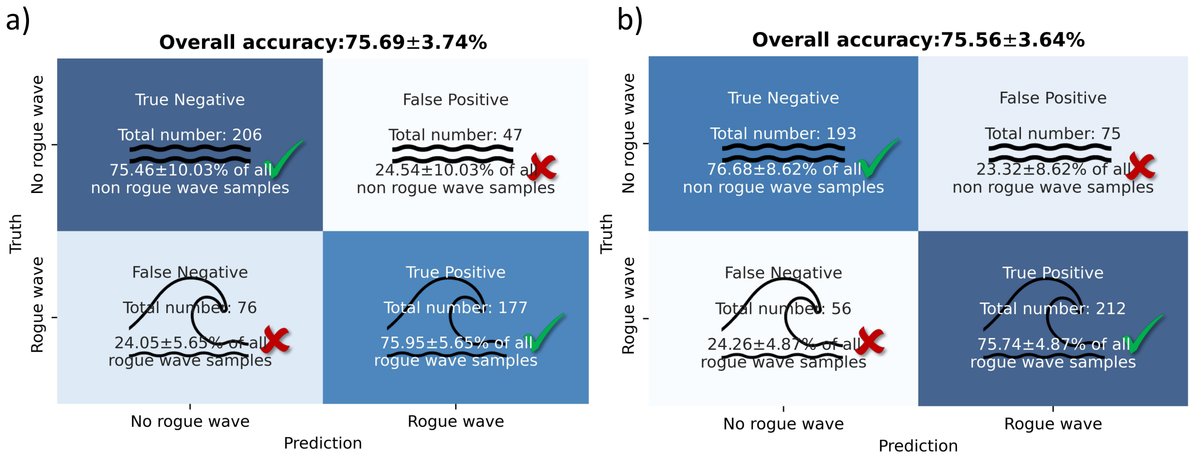

Figure S.2: Extrapolation of the LSTM network. Predictions with 95% confidence intervals from the neural network: a) Results for Buoy 132 located in shallow water (15m depth) of the coast of Jacksonville (Florida). b) Results for Buoy 166 remotely located in the Pacific in more than 4000 m water depth.

S.3 Rogue Wave Characteristics

Table S.1: Overview over the rogue waves extracted from the buoy measurements [36].

Buoy	Water	Number of rogue waves			Max. wave height		Max. wave crest height	
number	depth	$\frac{H}{H_s} > 2.2$	$\frac{H}{H_s} > 2$	$\frac{\eta_c}{H_s} > 1.25$	$\max(H)$	$\max\left(\frac{H}{H_s}\right)$	$\max(\eta_c)$	$\max\left(\frac{\eta_c}{H_s}\right)$
[−]	[m]	[−]	[−]	[−]	[m]	[−]	[m]	[−]
028	387.0	242	2615	380	9.35	2.62	5.49	1.52
029	550.0	466	4567	518	39.1	4.31	20.26	2.23
036	41.15	512	4667	516	16.8	2.77	9.73	1.61
043	20.0	133	1553	307	6.14	3.76	3.47	2.68
045	238.0	297	2981	323	10.62	2.65	6.4	1.55
067	274.32	331	3071	434	15.54	2.64	8.36	1.63
071	546.2	363	3681	369	20.45	2.66	10.29	1.59
076	27.43	373	3836	551	11.73	2.75	7.28	1.65
081	53.0	0	0	0	0	0	0	0
087	35.0	0	0	0	0	0	0	0
088	55.0	0	0	0	0	0	0	0
089	55.0	0	0	0	0	0	0	0
090	61.0	0	0	0	0	0	0	0
091	186.0	40	377	46	7.48	2.69	3.91	1.46
092	476.52	375	3590	423	8.13	2.65	4.34	1.53
093	192.0	135	1369	160	9.45	2.56	5.22	1.44
094	345.0	337	3030	330	24.08	3.43	14.23	2.03
095	181.1	71	772	81	6.58	2.54	3.87	1.43
096	370.0	195	1947	183	7.12	2.55	4.27	1.5
097	120.0	0	3	0	4.35	2.15	2.28	1.13
098	85.0	414	3908	549	11.21	2.83	6.18	1.55
099	100.0	0	4	0	3.21	2.14	1.64	1.11
100	571.96	272	3087	275	9.79	2.65	5.3	1.62
101	31.7	30	311	36	6.55	2.48	4.39	1.42
102	365.0	32	285	49	6.07	2.77	3.48	1.43
103	20.0	6	39	6	3.59	2.32	1.85	1.42
104	20.0	7	45	6	4.69	2.33	2.36	1.34
105	20.0	3	51	7	2.75	2.3	1.41	1.39
106	200.0	372	3725	352	12.23	4.87	6.87	2.84
107	182.57	273	2565	303	7.21	2.57	3.86	1.52
109	80.0	2	32	2	5.24	2.4	2.69	1.38
111	114.0	236	2645	394	7.19	2.65	4.16	1.75
113	35.0	9	115	16	5.19	2.39	2.94	1.37
114	35.0	16	145	14	4.52	2.35	2.4	1.37
115	20.24	24	193	29	8.75	2.5	5.3	1.49
116	27.43	29	243	23	4.46	2.59	2.55	1.46
117	20.0	5	55	5	4.76	2.33	2.49	1.47
118	20.0	13	99	25	3.88	2.45	2.23	1.65
121	200.0	386	4015	493	13.93	3.04	7.03	1.75
122	100.0	20	190	18	5.63	2.7	2.89	1.43
123	32.0	1	22	0	2.89	2.22	1.58	1.17
124	45.11	11	185	16	4.5	2.41	2.54	1.34
125	20.0	3	54	15	2.78	2.3	1.79	1.37
126	204.82	10	111	6	10.56	2.42	5.62	1.34
128	40.0	204	1975	164	13.49	2.65	7.74	1.48
129	18.5	2	20	4	3.06	2.26	1.73	1.33
130	20.0	17	114	17	3.13	2.36	1.69	1.51
131	21.5	10	144	15	3.51	2.42	1.87	1.39
132	15.24	253	2409	622	11.3	3.81	7.38	1.9
133	180.0	29	225	15	8.84	2.5	5.01	1.36
134	16.49	241	2410	744	6.87	3.13	4.67	1.63
135	200.0	3	12	3	10.24	2.41	5.81	1.42
136	50.0	3	41	3	5.98	2.27	3.2	1.32

Table S.1: Overview over the rogue waves extracted from the buoy measurements [36].

Buoy number	Water depth	Number of rogue waves			Max. wave height		Max. wave crest height	
		$\frac{H}{H_s} > 2.2$	$\frac{H}{H_s} > 2$	$\frac{\eta_c}{H_s} > 1.25$	$\max(H)$	$\max\left(\frac{H}{H_s}\right)$	$\max(\eta_c)$	$\max\left(\frac{\eta_c}{H_s}\right)$
[−]	[m]	[−]	[−]	[−]	[m]	[−]	[m]	[−]
138	105.0	49	408	76	10.86	2.53	6.75	1.47
139	185.0	406	3463	399	17.23	2.9	10.0	1.73
141	20.5	4	94	26	5.58	2.35	3.39	1.48
142	17.07	352	2353	1221	17.82	5.03	9.57	3.52
143	9.8	412	2270	877	24.17	5.12	12.14	2.69
144	93.88	607	4243	816	10.61	4.89	6.24	3.44
146	201.0	127	1336	177	7.77	2.99	4.64	1.81
147	15.0	243	2531	650	23.54	3.3	11.93	2.23
148	13.5	1	18	5	3.12	2.2	1.8	1.43
149	11.0	2	24	9	2.95	2.43	1.93	1.39
150	16.0	227	2372	667	6.95	2.6	4.27	1.6
151	21.0	5	39	11	3.98	2.31	2.08	1.43
152	23.5	3	21	6	4.78	2.33	2.65	1.42
154	50.29	282	2567	406	15.16	2.69	9.35	1.67
155	20.88	76	788	132	6.29	2.46	3.5	1.52
156	168.25	208	2024	193	10.98	2.63	6.31	1.53
157	369.0	271	2331	259	17.09	4.03	9.05	2.88
158	17.8	301	2892	315	7.87	2.76	5.41	1.66
160	80.0	365	3134	568	15.39	2.92	8.75	1.74
161	20.12	37	425	54	5.99	2.43	3.18	1.46
162	24.69	304	2648	521	24.51	3.19	14.54	2.06
163	553.0	168	1313	192	8.09	2.83	4.49	1.59
164	3150.0	16	175	35	10.04	2.4	5.1	1.54
165	300.0	63	823	105	8.13	2.41	4.39	1.53
166	4252.0	268	2425	319	34.66	3.57	17.52	1.8
167	1571.0	57	551	87	11.35	2.47	6.33	1.51
168	110.0	331	3266	250	21.55	2.79	12.72	1.48
169	19.81	11	122	30	4.46	2.39	2.63	1.4
170	177.0	37	295	74	16.66	3.08	9.32	2.05
171	49.0	25	357	53	13.74	2.43	7.65	1.49
172	22.2	19	178	29	6.57	4.05	4.88	3.01
174	230.0	38	249	58	17.1	2.65	9.84	1.71
176	53.0	37	278	35	10.44	2.46	5.43	1.41
179	181.36	222	2060	242	26.79	3.2	14.62	1.75
180	53.0	48	406	53	9.43	2.57	4.77	1.57
181	32.91	162	1847	246	9.1	2.87	4.85	1.57
182	1737.36	7	173	31	6.37	2.39	3.68	1.47
184	548.0	0	14	2	2.39	2.31	1.49	1.24
185	1463.0	246	2202	228	20.48	3.61	10.92	1.92
186	12.5	90	842	315	9.13	2.56	5.77	1.63
187	200.0	141	1263	186	10.59	2.71	5.42	1.52
188	345.0	244	2283	170	11.12	2.59	5.96	1.61
189	55.0	81	762	79	9.83	2.57	5.34	1.43
190	13.2	36	341	100	5.72	2.48	3.58	1.64
191	1049.8	201	2303	248	10.57	2.55	6.67	1.44
192	18.29	175	1659	385	13.41	2.6	8.63	1.55
193	678.0	32	302	36	7.07	2.46	4.03	1.37
194	23.8	66	710	195	8.59	2.59	4.6	1.6
195	71.0	34	251	37	22.57	3.23	12.24	1.75
196	500.0	197	1603	183	12.29	2.55	6.52	1.64
197	487.68	194	1832	164	16.25	2.61	8.53	1.44
198	79.0	199	1855	210	9.46	2.66	5.6	1.62
200	12.8	175	1856	559	8.0	2.67	5.38	1.67
201	46.0	177	1759	195	9.54	2.67	5.25	1.72
202	200.0	90	830	113	12.44	2.5	6.93	1.45

Table S.1: Overview over the rogue waves extracted from the buoy measurements [36].

Buoy number [−]	Water depth [m]	Number of rogue waves $\frac{H}{H_s} > 2.2$ [−]	$\frac{H}{H_s} > 2$ [−]	$\frac{\eta_c}{H_s} > 1.25$ [−]	Max. wave height $\max(H)$ [m]	$\max\left(\frac{H}{H_s}\right)$ [−]	Max. wave crest height $\max(\eta_c)$ [m]	$\max\left(\frac{\eta_c}{H_s}\right)$ [−]
203	1890.0	104	1064	208	8.92	2.52	5.09	1.59
206	200.0	16	126	19	9.22	2.85	5.1	1.68
207	25.0	34	322	74	9.29	2.71	5.38	1.77
208	549.0	15	134	13	4.92	2.36	2.82	1.44
209	25.6	173	1643	417	10.81	2.67	6.44	1.62
210	26.82	89	586	137	10.38	2.63	5.91	1.95
211	48.77	11	71	9	3.98	2.35	2.38	1.35
212	710.0	20	198	29	5.14	2.59	2.79	1.44
213	66.0	121	1254	170	9.56	2.57	5.63	1.48
214	14.0	544	2214	696	8.63	4.46	5.86	2.45
215	23.25	117	1225	131	6.84	2.61	4.22	1.46
216	576.07	60	460	64	18.31	2.55	10.14	1.43
217	30.0	128	1250	331	14.49	2.5	9.07	1.59
218	33.56	0	0	0	0	0	0	0
219	97.0	0	3	0	3.41	2.08	1.76	1.21
220	588.6	96	1079	107	11.79	2.72	6.08	1.47
222	650.0	139	1269	131	14.02	3.4	8.23	1.99
223	17.92	0	5	0	3.36	2.05	1.85	1.2
224	18.29	76	642	180	8.52	2.49	5.75	1.61
225	80.0	126	1217	133	9.52	2.51	4.9	1.47
226	81.0	185	1734	220	11.65	2.64	6.93	1.47
227	41.0	34	253	65	6.42	2.45	3.91	1.49
228	7.32	31	238	111	4.68	2.45	3.1	1.4
229	984.0	3	17	6	7.04	2.36	4.38	1.29
231	87.0	34	296	36	14.29	2.47	7.9	1.55
232	45.0	37	299	32	18.73	2.54	10.26	1.44
233	35.0	104	975	183	4.84	2.5	2.65	1.48
234	905.0	17	170	38	7.17	2.35	3.85	1.51
235	130.0	16	254	32	10.96	2.67	5.98	1.56
236	85.95	101	870	123	14.78	2.72	8.21	1.47
237	167.64	20	97	24	3.11	2.57	1.7	1.6
238	280.0	65	723	63	6.84	2.39	3.87	1.43
239	235.0	35	388	48	4.3	2.47	2.25	1.39
240	7.92	203	796	323	4.7	4.06	2.91	2.35
241	18.28	39	297	65	7.99	2.53	4.84	1.57
242	100.0	7	41	13	2.14	2.65	1.36	1.37
243	21.0	59	587	114	12.65	2.51	7.91	1.61
244	99.06	120	983	161	9.04	3.33	4.77	1.84
245	13.0	13	184	58	3.91	2.32	2.45	1.61
246	19.81	2	26	6	2.96	2.22	1.63	1.38
247	30.48	38	268	60	9.17	3.22	5.3	1.68
252	18.9	0	0	0	0	0	0	0
254	19.0	0	0	0	0	0	0	0
255	21.0	16	139	30	7.64	2.77	4.62	1.8
261	20.12	0	3	2	3.73	2.1	2.46	1.3
430	25.3	244	2422	470	12.76	3.0	6.87	1.93
431	255.0	161	566	184	7.36	3.17	3.69	1.91
432	530.0	21	183	55	6.53	2.68	3.45	1.84
433	18.14	186	1691	402	10.46	2.65	6.5	1.73
